# Shyness and self-consistency and congruence among Chinese adolescents: mediating role of social comparison orientation and moderating role of self-focused attention

**DOI:** 10.3389/fpsyg.2024.1418123

**Published:** 2024-07-09

**Authors:** Yang Yu, Hong Sun

**Affiliations:** College of Teacher Education, Taishan University, Shandong, Tai'an, China

**Keywords:** shyness, social comparison orientation, self-focused attention, self-consistency and congruence, Chinese adolescents

## Abstract

During the critical period of personality shaping and self-development, adolescents face unique challenges and opportunities. This study, based on Cognitive-Behavioral Theory, explored the relationship between shyness and self-consistency and congruence (hereinafter referred to as SCC), as well as its underlying mechanisms. Through a questionnaire survey on shyness, social comparison orientation, self-focused attention, and SCC among 984 adolescents, the results revealed that (1) Adolescent shyness negatively predicted SCC. (2) Social comparison orientation partially mediated the relationship between shyness and SCC. (3) Self-focused attention moderated the direct pathway of this mediation process, where a high level of self-focused attention exacerbated the negative impact of shyness on SCC. These findings offered a new perspective on understanding SCC and underscored the importance of addressing the information processing mechanisms of social comparison orientation and self-focused attention among shy adolescents in interventions aimed at promoting their psychological harmony and healthy growth.

## 1 Introduction

Shyness refers to the withdrawn and avoidant behavior exhibited by individuals in social or evaluative situations, typically accompanied by emotions such as tension and unease (Coplan et al., 2004). As a temperamental trait, shyness exhibits moderate stability from early childhood to adolescence (Coplan et al., 2004; Karevold et al., 2012), but is also influenced by environmental factors such as parental and peer relationships (Coplan et al., 2008; Yang et al., 2021). During adolescence, shyness is closely associated with internalizing problems (e.g., anxiety, depression), peer difficulties (e.g., rejection, victimization), and school challenges (e.g., academic procrastination, poorer academic performance) (Coplan et al., 2013; An and Eggum-Wilkens, 2021; Yang et al., 2021; Sun et al., 2024). For instance, Yang et al. (2015) pointed out that shyness-sensitivity has significant negative impacts on the social, school, and psychological adjustment of urban Chinese children. Over time, the negative impacts of shyness-sensitivity are particularly notable in peer preference and feelings of loneliness. An and Eggum-Wilkens (2021) conducted a longitudinal study which found that shyness exhibited by Chinese adolescents among familiar peers is significantly related to various maladjustments, especially academic and peer difficulties. Conversely, shyness in unfamiliar peer and formal situations is mainly associated with internalizing problems. These negative impacts create challenges for adolescents in harmonizing internal needs and external behaviors, making it difficult to form stable self-identity, thus disrupting SCC. However, the relationship between shyness and SCC, as well as its mechanisms, have not yet been thoroughly explored.

Cognitive-Behavioral Theory (CBT) emphasizes the interaction between individuals’ cognitive processes (i.e., thinking patterns), emotions, and behaviors, positing that emotional and behavioral problems are primarily determined by these cognitive processes, such as negative automatic thoughts (Kazantzis et al., 2018). This theory effectively explains the process by which shyness impacts SCC: shy individuals often possess negative automatic thoughts and cognitive biases in social situations, for example, believing that others hold negative views of them. Such negative cognitions lead to feelings of insecurity and anxiety (Coplan et al., 2013; Geng et al., 2021). To cope with this unease, shy adolescents frequently seek information from their surroundings, attempting to evaluate their social performance through social comparison, which undoubtedly increases their social comparison orientation (Festinger, 1954). At the same time, heightened self-focused attention causes shy adolescents to become more immersed in negative automatic thoughts and emotional responses, forming a difficult-to-escape negative cycle (Mor and Winquist, 2002; Kashdan and Roberts, 2004). They continually introspect and self-evaluate, magnifying the negative aspects of their cognitions, leading to stronger anxiety and feelings of inferiority. These emotions, in turn, reinforce their avoidant behavior, causing them to withdraw further in social interactions, reducing their opportunities for positive social experiences, and thus exacerbating internal conflict and dissatisfaction. This process collectively constitutes significant internal risk factors for the impact of shyness on SCC. Therefore, this study aims to comprehensively explore the relationship between shyness and SCC, as well as the roles of social comparison orientation and self-focused attention, to clarify this issue and provide targeted recommendations for promoting the development of SCC in adolescents.

### 1.1 Shyness and SCC

SCC refers to the alignment between the self and one’s experiences (or performance and perceptions) (Wang, 1994). It is not only a core pursuit in the Chinese cultural concept of harmony between humans and nature but also one of the most important concepts in Rogers’ personality theory. Although there is currently a lack of direct research on the relationship between shyness and SCC, related theoretical and empirical studies indicate a close correlation between the two.

Firstly, according to Asendorpf (1990), shy individuals often experience an approach-avoidance motivational conflict in social situations. This conflict arises from their strong need for social interaction and their fear of potential negative outcomes. Specifically, shy individuals desire social engagement, but they avoid it due to fears of rejection and criticism, resulting in a discrepancy between their inner expectations and actual behaviors. Subsequent research has confirmed the approach-avoidance motivational conflict. For example, Coplan et al. (2004) pointed out that shy children experience a conflict between approach and avoidance in social situations. They may want to interact with peers, but this desire is suppressed by social fear and anxiety. This motivational conflict is a significant cause of negative emotions, low self-worth, and difficulties in peer relationships (Coplan et al., 2013). From this perspective, shyness may be an important predictor of self-inconsistency in adolescents.

However, recent research has questioned the approach-avoidance motivational conflict in shy individuals. For instance, Hassan et al. (2021) proposed that the combination of a high Behavioral Inhibition System (BIS) and a low Behavioral Activation System (BAS) is closely related to the development of shyness, suggesting that shy individuals are primarily driven by avoidance motivation. While this avoidance behavior may reduce their social anxiety in short term, it may cause them to miss important and unique opportunities as they enter adolescence. Peers become increasingly influential in their social, emotional, cognitive, and moral development during this period. The growing social pressure and expectations present significant challenges for shy adolescents, potentially leading to internal conflicts and affecting their SCC. In summary, whether driven by approach-avoidance conflict or primarily by avoidance motivation, both can lead to self-inconsistency in shy adolescents.

Secondly, Self-Presentation Theory (Schlenker and Leary, 1982; Burke and Ruppel, 2015) emphasizes that individuals strive to project and maintain a self-image that facilitates the achievement of their goals during social interactions, rather than always presenting their true selves. Shy individuals attempt to present a self-image in social activities that gains acceptance and appreciation from others (Scott, 2007). However, they often feel that they lack effective social skills and find it difficult to successfully manage their self-image (Matsushima et al., 2000). This cognitive and emotional conflict frequently puts them in a state of anxiety and confusion, leading to a gap between self-perception and the ideal or actual self (Fullwood et al., 2020), resulting in self-inconsistency. For example, the study by Bober et al. (2021) indicated that negative self-statements in self-presentation significantly increased the risk of lowered self-esteem among shy individuals. Similarly, Geng et al. (2021) found that shy individuals often have lower core self-evaluations and a sense of security, indicating conflicts in self-perception and self-acceptance. Based on the above analysis, this study hypothesized that shyness predicts SCC (Hypothesis 1).

### 1.2 The mediating effect of social comparison orientation

According to Festinger’s (1954) Social Comparison Theory, individuals tend to evaluate their own social status by comparing themselves with others. Adolescents are in a developmental stage of actively exploring themselves and shaping their identities, where social comparison orientation plays a significant role (Krayer et al., 2008).

On one hand, based on the research of Asendorpf (1990) and Self-Presentation Theory, shy adolescents, whether worried about encountering criticism or rejection or desiring to present a favorable self-image, may frequently engage in self-reflection and evaluation. This process often requires external feedback through social comparison, yet the impact of both the process and its outcomes is generally negative (Santor and Yazbek, 2006; Vogel et al., 2015). Therefore, shyness may predict social comparison orientation.

On the other hand, shy adolescents, due to a lack of confidence in social activities, rely more on social comparison than on their own intrinsic motivation to obtain and confirm external information. This leads to a lack of SCC among shy adolescents, meaning there is a significant gap and conflict between their cognition of self-worth and social status and their internal ideal self (Vogel et al., 2015). Therefore, adolescents with a high social comparison orientation may exacerbate this sense of self-inconsistency.

Recent empirical research further emphasized the important role of social comparison orientation in shaping adolescents’ self-perception and emotional regulation. For example, Ruan et al. (2023) found that lower self-acceptance and higher levels of social comparison orientation are significantly related to an increase in depressive and anxiety symptoms among adolescents, indicating that social comparison orientation plays a key mediating role between self-acceptance and depression/anxiety. Therefore, the self-incongruence of shy adolescents may stem from their high social comparison orientation. Based on the above analysis, this study proposed Hypothesis 2: Social comparison orientation mediates the relationship between shyness and SCC.

### 1.3 The moderating effect of self-focused attention

Shyness affects adolescent SCC through social comparison orientation, but not all adolescents are impacted to the same extent, suggesting the possibility of moderating factors. This study introduced “self-focused attention” as a moderating variable to explore its role in the “Shyness → Social Comparison Orientation→ SCC” process.

Self-focused attention refers to the process of concentrating attention on one’s internal feelings, thoughts, attitudes, and behaviors (Ingram, 1990). According to Self-Focused Attention Theory, the level of an individual’s self-focused attention significantly affects their self-evaluation and emotional experiences. High levels of self-focused attention may lead individuals to excessively focus on the standards for evaluating self-worth and external feedback, negatively impacting their self-cognition and emotional state (Ingram, 1990; Mor and Winquist, 2002). Conversely, individuals with lower levels of self-focused attention may be less dependent on self-evaluation and external judgments in social interactions, thus exhibiting a more relaxed and comfortable attitude (Alm, 2007).

In social situations, shy adolescents may exhibit apparent social deficits due to their concerns about criticism or rejection, and an excessive focus on presenting a favorable self-image. If combined with high levels of self-focused attention, this may lead them to overly emphasize self-presentation in school and social life, falling into a cycle of over-reflection and self-evaluation, thereby exacerbating negative emotions such as confusion, unease, and stress (Ingram, 1990; Mor and Winquist, 2002; Kashdan and Roberts, 2004). This results in more difficulties in handling self-identity and emotional management, failing to meet individuals’ internal needs (Blöte et al., 2019), and ultimately leading to self-incongruence. On the contrary, if shy adolescents have lower levels of self-focused attention, they may be relatively relaxed in social interactions, less concerned with right and wrong, thus less negatively affecting their SCC. Therefore, self-focused attention may serve as a moderating factor between shyness and SCC, suggesting that as the level of self-focused attention increases, the SCC level of shy individuals may decrease.

An earlier study observed that under conditions of low self-focused attention, the association between social anxiety and adverse social adaptations, such as fear, facial blushing, negative cognition, and social avoidance behaviors, was weakened (Wells et al., 1995). Recent studies have also found that reducing self-focused attention levels helps alleviate negative experiences related to body image (including appearance satisfaction and personal attractiveness) and self-esteem (Barnier and Collison, 2019). Based on the above analysis, this study proposed that self-focused attention can enhance the predictive effect of shyness on SCC, that is, self-focused attention moderates the direct pathway in the “Shyness → Social Comparison Orientation→ SCC” process (Hypothesis 3).

### 1.4 The present study

**Figure 1:**
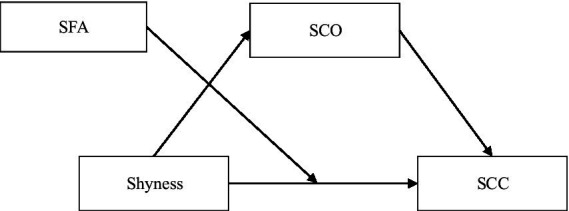
Conceptual model. SCO, social comparison orientation; SFA, self-focused attention; SCC, self-consistency and congruence.

In summary, this study aimed to test the following hypotheses (see Figure 1): (1) Adolescent shyness negatively predicts SCC; (2) Social comparison orientation mediates the relationship between shyness and SCC; (3) Self-focused attention moderates the relationship between shyness and SCC.

## 2 Methods

### 2.1 Participants and procedures

The study was conducted with randomly selected 20 classes from five regular middle schools in Shandong Province, encompassing grades one through three. We distributed 1,000 questionnaires, receiving 984 valid responses, with a 98.4% response rate. Valid responses were 339, 349, and 296 for grade one, two, and three, respectively. Participants included 483 boys and 501 girls; 462 only children and 522 with siblings, 514 from urban and 480 from rural areas. The average age was 14.5 years (SD = ±1.2). Data collection was completed in 45-min sessions by trained educators, following a standardized protocol. Ethical approval was granted by the Tai Shan University Ethics Committee, with informed consent from students, parents, and teachers.

### 2.2 Measures

#### 2.2.1 Shyness

The Revised Cheek and Buss Shyness Scale (RCBS; Cheek, 1983) was used to assessed adolescent shyness. This scale consists of 13 items (samples like “I am quite poor in social situations”) and has been widely used in Chinese students (Geng et al., 2021; Sun et al., 2024). Responses are provided using 5-point scale ranging from 1 (not all) to 5 (very) and a higher score indicates higher shyness. In this study, Cronbach’s *α* was 0.76.

#### 2.2.2 Social comparison orientation

Social comparison orientation was assessed using the Chinese version (Wang et al., 2006) of Iowa-Netherlands Comparison Orientation Measure developed by Gibbons and Buunk (1999). The measure includes 11 items (e. g. “I am always very interested in noticing how I do things differently from others”) and uses a 5-point scale. Higher scores indicate increased social comparison orientation levels. The measure is applicable to Chinese students and demonstrates good validity and reliability (Zhang et al., 2023). In this study, Cronbach’s α was 0.71.

#### 2.2.3 Self-focused attention

Self-focused attention was measured by Self-focused Attention Scale (SFAS), which was developed by Kiropoulos and Klimidis (2006) and refined by Xiao (2010). It includes 17 items (sample item “I care a lot about the way I present myself physically”) using a 5-point scale (score range: 17–85), where higher scores indicate greater self-focused attention. The scale is suitable for Chinese students (Ding et al., 2021). In this study, Cronbach’s α was 0.74.

#### 2.2.4 Self-consistency and congruence

We employed the Self-Consistency and Congruence Scale (Wang, 1994) to measure SCC. This scale, extensively utilized among Chinese students (Ge et al., 2021), comprises 35 items. A sample item is, “People around me often perceive a certain contradiction in my self-perception,” which is evaluated using a five-point scale with a score range of 35–175. Conventionally, higher scores indicate lower levels of SCC. However, in this study, we inverted the scoring, deducting the original scores from the overall points, to bring uniformity. Thus, following this inversion, higher scores denote higher levels of SCC. The Cronbach’s α for this study was 0.75.

### 2.3 Statistical analysis

Data were organized and analyzed using SPSS 26.0 and SPSS macro PROCESS 4.0 (Hayes, 2013) for descriptive statistics, two-way ANOVA, mediation, and moderation tests. Applying a 95% confidence interval, the mediation and moderated mediation effects were evaluated employing 5,000 bootstrap samples. Prior to conducting analyses, all quantitative variables underwent standardization. Harman’s single-factor test was employed to assess common method bias. An exploratory factor analysis of all items for shyness, social comparison orientation, self-focused attention, and SCC revealed that the variance explained by the first factor was 10.07%, which is below the critical threshold of 40%, indicating no significant common method bias.

## 3 Results

### 3.1 Descriptive statistics and correlations

Table 1 presents the descriptive statistics and correlation matrix. Shyness was negatively connected to SCC and positively with social comparison orientation (SCO). SCC was negatively correlated with social comparison orientation (SCO) and self-focused attention (SFA).

**Table 1:** Descriptive statistics and intercorrelations between variables.

	Variables	*M*	*SD*	1	2	3	4
1	Shyness	34.270	8.547	1.000			
2	SCO	33.890	6.162	0.115^**^	1.000		
3	SFA	57.572	9.579	0.023	0.459^**^	1.000	
4	SCC	64.624	14.123	−0.328^**^	−0.355^**^	−0.406^**^	1.000

^*^p < 0.05 (two-tailed). ^**^p < 0.01 (two-tailed). SCO, social comparison orientation; SFA, self-focused attention; SCC, self-consistency and congruence. (*N* = 984).

### 3.2 Two-way ANOVA

We conducted two-way ANOVAs to examine the effects of grade (Grades 1, 2, and 3) and gender (male, female) on four variables: shyness, SCO, SFA, and SCC (see Table 2). The results indicated significant main effects of gender on shyness, SCO and SFA, with girls scoring higher than boys on these three variables. There were no significant main effects of grade on any of the variables, nor were there significant interaction effects between grade and gender on any of the variables.

**Table 2:** Results of two-way ANOVAs on SCC, SCO, SFA, and shyness.

Source	Shyness (*F, η^2^*)	SCO (*F, η^2^*)	SFA (*F, η^2^*)	SCC (*F, η^2^*)
Grade	1.274 (0.003)	0.288 (0.001)	0.056 (0.000)	1.030 (0.002)
Gender	13.529^**^ (0.014)	14.376^**^ (0.014)	13.243^**^ (0.013)	0.068 (0.000)
Grade * Gender	0.041 (0.000)	0.943 (0.002)	1.216 (0.002)	0.255 (0.001)

^*^p < 0.05 (two-tailed). ^**^p < 0.01 (two-tailed). SCO, social comparison orientation; SFA, self-focused attention; SCC, self-consistency and congruence.

### 3.3 Mediation analysis

Using PROCESS Model 4, we examined the mediation effect of social comparison orientation between shyness and SCC. The results suggested that shyness positively predicted social comparison orientation (*β* = 0.115, *SE* = 0.032, *t* = 3.632, *p* < 0.001) and negatively predicted SCC (*β* = −0.291, *SE* = 0.029, *t* = −10.198, *p* < 0.001). Social comparison orientation also negatively predicted SCC (*β* = −0.321, *SE* = 0.029, *t* = −11.238, *p* < 0.001) (see Table 3). The indirect effect was −0.04 with a 95% confidence interval [−0.061, −0.014], not containing zero, indicating that social comparison orientation served as a partial mediator.

**Table 3:** Bootstrapping effect for the mediation model.

Path	*b*	Boot *SE*	Boot 95%*CI*		Effect (%)
			Lower	Upper	
Indirect effect	−0.037	0.012	−0.061	−0.014	11.28%
Direct effect	−0.291	0.029	−0.347	−0.235	88.72%
Total effect	−0.328	0.030	−0.388	−0.269	100%

Bootstrap sample size = 5,000; CI = confidence interval. (*N* = 984).

### 3.4 Moderated mediation analysis

**Figure 2:**
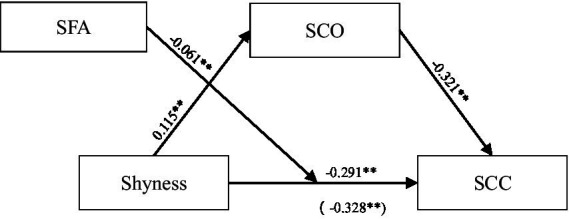
Path coefficient test results. SCO, social comparison orientation; SFA, self-focused attention; SCC, self-consistency and congruence.

Using PROCESS Model 5, We tested the moderating effect of self-focused attention (SFA). The results (see Table 4) indicated a significant negative interaction effect of shyness and self-focused attention (SFA) on SCC (*β* = −0.061, *SE* = 0.026, *t* = −2.358, *p* < 0.05), suggesting that self-focused attention (SFA) negatively moderated the relationship between shyness and SCC. The coefficients of each pathway were presented in Figure 2, with total effects in parentheses.

**Table 4:** Conditional process analysis (*N* = 1,279).

	*β*	*SE*	*t*	95% *CI*
**Mediator variable model predicting SCO**				
Shyness	0.115	0.032	3.632^**^	[0.053, 0.177]
**Dependent variable model predicting SCC**				
Shyness	−0.294	0.027	−10.797^**^	[−0.347, −0.240]
SCO	−0.175	0.030	−5.746^**^	[−0.235, −0.115]
SFA	−0.324	0.030	−10.683^**^	[−0.383, −0.264]
Shyness ×SFA	−0.061	0.026	−2.358^*^	[−0.111, −0.010]
**Conditional effect**	Effect	Boot *SE*	Boot *LLCI*	Boot *ULCI*
*M* − 1*SD*	−0.233	0.039	−0.311	−0.156
*M*	−0.294	0.027	−0.347	−0.240
*M* + 1*SD*	−0.355	0.035	−0.424	−0.285

^*^p < 0.05 (two-tailed). ^**^p < 0.01 (two-tailed). Bootstrap sample size = 5,000. SCO, social comparison orientation; SFA, self-focused attention; SCC, self-consistency and congruence. CI = confidence interval. LL = low limit. UL = upper limit.

**Figure 3:**
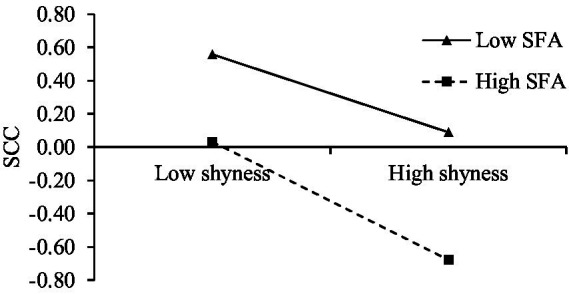
Self-focused attention moderated the relationship between shyness and SCC. SFA, self-focused attention; SCC, self-consistency and congruence.

Simple slope analyses were conducted based on groups divided by M ± 1SD of self-focused attention (SFA). The results (see Figure 3) showed that at low levels of self-focused attention (SFA), shyness negatively predicts SCC (*β* = −0.233, *SE* = 0.039, *t* = −5.905, *p* < 0.001); at high levels of self-focused attention (SFA), this predictive effect was enhanced (*β* = −0.355, *SE* = 0.035, *t* = −10.036, *p* < 0.001). This indicated that as the level of self-focused attention (SFA) increases, the negative predictive effect of shyness on SCC strengthened.

## 4 Discussion

This study, guided by Cognitive-Behavioral Theory and Self-Presentation Theory, revealed the relationship and mechanisms between shyness and SCC. The process by which shy individuals experience dissonance in SCC can be understood as a “bidirectional information acquisition” process. On one hand, shy adolescents’ social comparison orientation (mediating role) leaded them to acquire and process external information, increasing internal conflict and anxiety, resulting in self-inconsistency. On the other hand, excessive self-focused attention (moderating effect) in shy adolescents may amplify their perception of internal discomfort and conflict, further reinforcing self-denial and reducing SCC. This provided a new perspective for understanding the relationship between shyness and SCC, suggesting that in practice, attention should be paid to the shy individuals’ acquisition of both internal and external information to more effectively explore and develop suitable intervention measures.

### 4.1 Demographic variable analysis

This study found that adolescent girls exhibited significantly higher levels of shyness, social comparison orientation, and self-focused attention than boys: (1) Adolescent girls showed significantly higher levels of shyness compared to boys, consistent with previous research findings (Gazelle et al., 2014). This suggested that due to physiological and psychological changes, adolescent girls had deeper emotional experiences and were more sensitive to social interactions. Although boys exhibited lower levels of shyness, they might have faced greater challenges in social adaptation and internalizing problems due to shyness (Gazelle et al., 2014; Rubin and Barstead, 2014), which is related to the expectation for males to display social confidence and dominance. (2) Girls had significantly higher social comparison orientation than boys, indicating that they were more inclined to evaluate their social status and performance by comparing themselves to others, possibly due to higher societal and cultural standards for appearance and behavior in females (Vogel et al., 2015). (3) Girls also exhibited significantly higher levels of self-focused attention than boys, indicating a greater tendency for self-reflection and introspection, which was associated with their higher emotional sensitivity and emotional processing ability (Nolen-Hoeksema and Aldao, 2011). High levels of self-focused attention might have led adolescent girls to ruminate more when facing stress, increasing the risk of anxiety and depression (Papadakis et al., 2006). These findings suggested the need for gender-specific educational and psychological interventions.

The study also found that grade level did not have a significant impact on these variables, nor did the interaction between gender and grade level. This indicated that these traits are relatively stable during adolescence and do not significantly change over a short period due to grade level. This highlights the importance of early intervention. Additionally, the effects of gender and grade level on these variables were independent, with consistent gender differences observed across all grade levels, and grade level changes did not significantly affect these gender differences. This suggests that intervention measures can be optimized separately for different gender and grade groups.

### 4.2 Shyness and SCC

This study found that shyness significantly negatively predicts SCC in adolescents, highlighting the challenges shy adolescents face in self-acceptance, self-growth, and achieving internal psychological balance. These results not only aligned with Self-Presentation Theory (Schlenker and Leary, 1982; Burke and Ruppel, 2015) but also reaffirmed the negative impact of shyness on adolescents (An and Eggum-Wilkens, 2021; Yang et al., 2021; Sun et al., 2024).

SCC is an important indicator of psychological health and also a critical factor in the emergence of various psychological issues. Existing researches have widely explored the associations between shyness and psychological problems such as low self-esteem, depression, and anxiety, with efforts directed toward intervention (Coplan et al., 2013; Staring et al., 2016; Geng et al., 2021). However, these studies overlooked the underlying causes of psychological health issues in shy adolescents. By focusing on the link between shyness and SCC, this research not only deepens the understanding of the relationship between shyness and psychological health but also uncovers the root causes of psychological health issues in adolescents, thereby facilitating the exploration of causes and the integration of intervention strategies for psychological health problems.

### 4.3 Mediating effect of social comparison orientation

This study revealed that shyness not only directly predicts SCC but also indirectly affects SCC through social comparison orientation, further corroborating Festinger’s (1954) Social Comparison Theory and aligning with the findings of Ruan et al. (2023). Shy adolescents tended to engage in social comparison for self-positioning, making them susceptible to negative self-evaluations, thereby damaging SCC.

On one hand, shy adolescents are highly sensitive to others’ evaluations, frequently engaging in social comparison and exhibiting excessive concern for external opinions. From a cognitive perspective, such comparison orientation leads to negative self-evaluations, especially when they perceive themselves as inferior in social performance or abilities. Emotionally, the negative self-evaluation triggered by social comparison intensifies shy adolescents’ feelings of insecurity, fear, and avoidance of social environments and interpersonal interactions.

On the other hand, social comparison orientation negatively predicted SCC. The foundation of SCC lies in an individual’s ability to balance internal needs with external social behavior norms. Individuals with high social comparison orientation often over-rely on external information, neglecting internal emotions and actual needs, leading to increased internal inconsistency. Previous researchers have also analyzed the negative impacts of social comparison orientation from various perspectives, including envy and dissatisfaction on social media, and discontent with body image (Tiggemann and McGill, 2004; Appel et al., 2016). Therefore, individuals highly engaged in social comparison face greater challenges in achieving SCC.

In summary, this study offered a new perspective on how shyness affects individuals’ psychological health and social adaptability through the mechanism of social comparison orientation, emphasizing the importance of reducing the negative impact of social comparison orientation to promote SCC and psychological well-being in shy adolescents.

### 4.4 Moderating effect of self-focused attention

Theoretical and empirical research indicated that self-focused attention significantly impacts individual social adaptation (Ingram, 1990; Wells et al., 1995; Mor and Winquist, 2002; Sun et al., 2024). Previous studies have explored the role of self-focused attention in psychological counseling and academic adjustment. This study expanded this domain by examining the moderating role of self-focused attention in the process of self-development, making an important exploration.

This study found that individuals with high levels of self-focused attention, shyness had a more significant negative impact on SCC. This finding supports the perspective of Self-Focused Attention Theory (Ingram, 1990; Mor and Winquist, 2002). The increase in self-focused attention not only intensified adolescents’ concerns and worries about their inadequacies but also limited their ability to fulfill self-development needs and establish positive interpersonal relationships through social interactions. Shyness, as a stable personality trait, restricted adolescents’ willingness and ability to actively participate in social activities, making it difficult for them to obtain necessary emotional support and positive feedback (Yang et al., 2021). The heightened self-focused attention exacerbated this issue, making adolescents more susceptible to the pressure of social adaptation. Therefore, for adolescents with high levels of self-focused attention, shyness is not only a personal burden but also an aggravation of their existing sense of self-incongruence, worsening their psychological discomfort.

Conversely, for adolescents with lower levels of self-focused attention, shyness had a lesser negative impact on their mental health (Alm, 2007; Hassan et al., 2020). This may be due to their less focus on personal inadequacies and external evaluations, thus alleviating the social anxiety and psychological pressure brought by shyness. This finding suggested that reducing the level of self-focused attention may be one of the effective ways to alleviate the sense of self-incongruence in shy adolescents, aligning with previous research findings that lowering self-focused attention levels can promote individual psychological health (Barnier and Collison, 2019). The results of this study validated the risk role of self-focused attention and also confirmed that the internal factors affecting individual SCC do not operate independently but are interconnected. The accumulation of risk factors (shyness compounded by self-focused attention) is more likely to lead to poorer developmental outcomes. Given that adolescents’ self-development matures with age, developmental risk factors should be highly concerned.

### 4.5 Cultural applicability and educational implications

Cultural background can influence individual psychological and behavioral responses to some extent, but the mechanisms and pathways through which shyness affected SCC may exhibit cross-cultural consistency.

Firstly, shyness impacted adolescents in both Eastern and Western cultural contexts. As a universally prevalent personality trait, shyness manifested as high sensitivity to social evaluation and avoidance of social situations in both cultural environments (Poole et al., 2020; Geng et al., 2021; Yang et al., 2021). The inherent characteristics of shyness caused adolescents to feel uneasy and inferior in the presence of others, thereby affecting their self-perception and self-worth. Thus, this negative impact was significant among adolescents across different cultural backgrounds.

Secondly, social comparison orientation was an important means for adolescents to assess their self-worth. In Eastern cultures, where collectivism is more prevalent, individuals are more inclined to seek self-identity and social recognition through social comparison (Yuki, 2023). In contrast, in Western cultures, where individualism dominates, individuals tend to define meaning through self-perception and self-worth, using social comparison more for self-enhancement and uniqueness affirmation (Markus and Kitayama, 1991; Baldwin and Mussweiler, 2018). Despite cultural differences, the core role of social comparison as a self-assessment mechanism was significant in both Eastern and Western cultures. Whether seeking collective recognition or personal achievement, social comparison orientation exacerbated the self-inconsistency among shy adolescents.

Moreover, high levels of self-focused attention amplified the negative impact of shyness on SCC. In a cultural environment where collectivism prevails, high levels of self-focused attention may lead individuals to be more concerned with others’ evaluations and expectations, thereby exacerbating the self-inconsistency caused by shyness. In a cultural context dominated by individualism, high levels of self-focused attention may lead individuals to place more emphasis on personal achievements and self-expression (Markus and Kitayama, 1991; Tsai, 2021), which also magnifies the negative impact of shyness on SCC. This moderated mechanism underscored the crucial role of internal self-monitoring in psychological adaptation. Regardless of how success and self-worth were defined by culture, higher levels of self-focused attention made shy adolescents more vulnerable, leading to psychological incongruence.

The results of this study have significant implications for educational practice:

Firstly, attention should be given to the levels of shyness in children. When shyness levels are high, regardless of the degree of self-focused attention, the level of SCC is low. It is important to identify and intervene in cases of shyness early, implementing various approaches such as mental health education and social skills training. This should be a collaborative effort among families, schools, and communities to establish a supportive and understanding environment. Through emotional support and positive feedback, shy adolescents can build confidence and SCC. As Rubin and Coplan (2010) pointed out, early identification and intervention of shy behavior are crucial for promoting children’s psychological health and social adaptation.

Secondly, it is important to address the negative impact of social comparison orientation. Vogel et al. (2015) found that high social comparison orientation is associated with lower self-esteem and higher levels of depression. This study further emphasizes the necessity of reducing social comparison orientation to improve adolescent mental health. Although systematic interventions for social comparison orientation are still relatively lacking, some researchers have made preliminary attempts. For example, Vimalakanthan et al. (2018) studied 120 female college students and found that fostering a compassionate mindset significantly reduced body dissatisfaction and restrictive eating behaviors, particularly for those who frequently engage in social comparison. This indicated that interventions for social comparison orientation are feasible and effective. Adolescence is a critical period of development, and fostering positive information acquisition methods is valuable for enhancing positive qualities.

Thirdly, reasonable self-focused attention should be emphasized. Self-focused attention not only reduces SCC but also amplifies the negative impact of shyness on SCC. Therefore, interventions should focus on reducing excessive self-focused attention in shy adolescents. This can be achieved in various ways. For example, Steele and Day (2018) found that reflective self-attention is positively correlated with leadership self-efficacy and self-reported leadership role development, whereas ruminative self-attention has a negative impact on leadership development. This suggests that guiding shy adolescents to engage in positive self-reflection can reduce ruminative self-focused attention, thereby improving their SCC. Additionally, group activities, team cooperation, and social interactions can shift attention from personal deficiencies to the external environment and the needs of others, enhancing self-identity and psychological satisfaction.

Finally, from a demographic analysis perspective, the results of this study have important practical implications for guiding adolescent education and psychological interventions: (1) Educational and psychological interventions for shyness and its related psychological variables should be tailored to different genders. For girls, interventions should focus on helping them manage shyness and high self-focused attention levels to reduce the risk of anxiety and depression. For boys, interventions should address the conflict between shyness and social expectations, alleviating social adaptation difficulties and internalization problems. (2) Since these traits are relatively stable during adolescence, early intervention is particularly important. Educators and mental health professionals should identify and support adolescents with high levels of shyness and self-focused attention early on, providing appropriate counseling and support to prevent anxiety and depression. (3) Interventions should be optimized for different gender and grade groups. Differentiated intervention strategies can more effectively meet the needs of various groups, promoting psychological health and social development in adolescents. In summary, by considering the influence of gender and grade, scientifically designed interventions can better help adolescents develop healthy self-perception and social skills, enhancing SCC.

### 4.6 Limitations and future directions

First, this study employed a cross-sectional design, which precludes causal inferences between variables. This limitation implies that the mediating effects of shyness on predicting SCC identified in this study should be viewed as preliminary theoretical hypotheses rather than definitive causal pathways. Nevertheless, based on existing theories and supporting literature, we believe these preliminary findings hold significant theoretical and practical implications. Future research should adopt longitudinal designs to further validate these hypotheses, clarify the causal relationships between variables, and elucidate their dynamic processes over time.

Second, in this study, we used the Revised Cheek and Buss Shyness Scale (RCBS; Cheek, 1983) to measure adolescents’ levels of shyness. Although the RCBS scale reflects the psychological motivations and behavioral responses of shy individuals in social situations, consistent with key assumptions of Asendorpf’s study, the Child Social Preference Questionnaire (CSPQ) provides a more detailed perspective on assessing approach and avoidance motivations of shyness (e.g., Coplan et al., 2013; Li et al., 2016). This detailed perspective helps to more accurately predict the impact of shyness on SCC. Therefore, to enhance the comprehensiveness and reliability of research findings, future studies should consider using multiple scales for cross-validation, thereby providing a more robust foundation for theoretical and empirical research in this field.

Third, it is necessary to distinguish between upward and downward comparisons. Upward and downward comparisons have different psychological and behavioral impacts on individuals. For instance, Buunk et al. (2005) found that students engaging in upward comparisons in the classroom tend to experience stronger negative emotions, such as inferiority and anxiety, which may further affect their SCC. Conversely, downward comparisons can make adolescents feel proud and downward temporal comparisons can stimulate their motivation for self-improvement (Gürel et al., 2022), thereby enhancing their sense of SCC. Consequently, downward comparisons can serve as a self-protection strategy after a threat, helping individuals restore their self-esteem (Michinov and Bavent, 2001). Future research should also explore the specific psychological mechanisms of upward and downward comparisons. For example, examining how upward comparisons affect SCC of shy individuals by increasing stress and anxiety, and how downward comparisons mitigate the negative impact of shyness on SCC by enhancing self-esteem and satisfaction. Understanding these mechanisms in-depth will aid in the development of more effective mental health intervention strategies.

Finally, cross-cultural research is necessary to deeply analyze the specific manifestations and differences in the mechanisms and pathways through which shyness impacts SCC. For instance, in collectivist cultures, social comparison orientation may focus more on group harmony and identification, while in individualist cultures, they may focus more on personal achievements and uniqueness (Markus and Kitayama, 1991). Xiao and Coplan (2021) found that although both Canadian and Chinese students automatically associate shyness with negative terms, the patterns of association in self-reports differ between the two countries. Thus, shy adolescents in different cultural contexts may experience and handle their negative emotions in different ways, further illustrating the impact of cultural background on SCC of shy adolescents. Future research could reveal the deep-seated cultural influences on the relationship between shyness and SCC, thereby developing culturally adaptive psychological interventions to more effectively promote adolescents’ mental health and SCC.

## Data availability statement

The original contributions presented in the study are included in the article/supplementary material, further inquiries can be directed to the corresponding author/s.

## Ethics statement

The studies involving humans were approved by the Tai Shan University Ethics Committee. The studies were conducted in accordance with the local legislation and institutional requirements. Written informed consent for participation in this study was provided by the participants’ legal guardians/next of kin.

## Author contributions

YY: Conceptualization, Funding acquisition, Project administration, Resources, Supervision, Validation, Visualization, Writing – original draft, Writing – review & editing. HS: Data curation, Formal analysis, Investigation, Methodology, Writing – original draft, Writing – review & editing.
